# Natural Windbreaks Sustain Bird Diversity in a Tea-Dominated Landscape

**DOI:** 10.1371/journal.pone.0070379

**Published:** 2013-07-29

**Authors:** Rachakonda Sreekar, Anand Mohan, Sandeep Das, Prerna Agarwal, Ramachandran Vivek

**Affiliations:** 1 Key Laboratory of Tropical Forest Ecology, Xishuangbanna Tropical Botanical Garden, Chinese Academy of Science, Mengla, Yunnan, China; 2 Department of Zoology, Fergusson College, Pune, Maharashtra, India; 3 Department of Zoology, Farook College, Kozhikode, Kerala, India; 4 Department of Biodiversity, M. E. S. Abasaheb Garware College, Pune, Maharashtra, India; 5 Ashoka Trust for Research in Ecology and the Environment (ATREE), Bangalore, Karnataka, India,; 6 University of the Chinese Academy of Sciences, Beijing, China; The Pennsylvania State University, United States of America

## Abstract

Windbreaks often form networks of forest habitats that improve connectivity and thus conserve biodiversity, but little is known of such effects in the tropics. We determined bird species richness and community composition in windbreaks composed of remnant native vegetation amongst tea plantations (natural windbreaks), and compared it with the surrounding primary forests. Fifty-one, ten-minute point counts were conducted in each habitat type over three days. Despite the limited sampling period, our bird inventories in both natural windbreaks and primary forests were nearly complete, as indicated by bootstrap true richness estimator. Bird species richness and abundance between primary forests and windbreaks were similar, however a difference in bird community composition was observed. Abundances of important functional groups such as frugivores and insectivores did not vary between habitat types but nectarivores were more abundant in windbreaks, potentially as a result of the use of windbreaks as traveling routes, foraging and nesting sites. This preliminary study suggests that natural windbreaks may be important habitats for the persistence of bird species in a production landscape. However, a better understanding of the required physical and compositional characteristics for windbreaks to sustain bird communities is needed for effective conservation management.

## Introduction

The expansion of production landscapes and the subsequent fragmentation of native habitat in the tropics can alter community assemblages and gene flow in isolated communities [1]. Landscape connectivity is therefore an important component for conservation efforts, which has led to an increased focus on remnant forests in plantations for population connectivity [2]. Windbreaks are linear strips of planted trees or retained remnant vegetation to protect commercial crops from wind-damage. Windbreaks can potentially act as corridors in deforested landscapes when they connect forest fragments, but the use of windbreaks by native animal communities is poorly known.

Functionally, birds are one of the most diverse groups of vertebrates [3], and evidences from previous studies indicate that bird functional groups respond differently to land use changes [4]. Each functional group is important for a particular ecological function: carnivores, granivores, frugivores, insectivores and nectarivores that provide pest predation, seed predation, seed dispersal, insect abundance regulation and pollination services, respectively. Numerous studies in tropical agroforests like cocoa and coffee plantations provide a sound foundation concerning the effects of habitat alteration on functional group composition (e.g., [5,6]), but other important habitat structures of a production landscape, including windbreaks, remain understudied. Preliminary studies show that birds may use windbreaks as corridors [7,8], but the responses of different functional groups to windbreaks remain poorly known.

Information about changes in species composition and functional diversity of birds in windbreaks is particularly important in biodiversity hotspots that have undergone extensive habitat fragmentation, such as the Western Ghats in southwestern India [9,10]. Several protected areas established in the remaining forest are a highly variegated mosaic of both natural and managed ecosystems [11]. Tea is one of the important plantation crops in the Western Ghats, occupying an area over 1100 km^2^ and producing more than 219,000 metric tons of dried tea annually [12-14]. Tea is planted throughout the Western Ghats between 400 and 2100m altitude [15]. Windbreaks in the tea plantations of the Western Ghats are linear strips of natural forest (natural windbreaks) or plantations of exotic species, such as Eucalyptus (planted windbreaks) that are maintained or planted to reduce crop damage by protecting the tea against wind damage and by reducing soil erosion [16].

Production landscapes containing natural habitats can harbour a substantial proportion of the regional bird species [17-20]. Natural windbreaks are one such habitat that may be critical for the persistence of birds in production landscapes. Hence, we examined the use of natural windbreaks by birds in a tropical production landscape by comparing the species richness and abundances of birds in windbreaks with that found in nearby primary forests. We further compare the species richness and abundances of important bird functional groups, and abundances of every Western Ghats endemic species across these landscape features.

## Methods

### Study site

**Figure 1:**
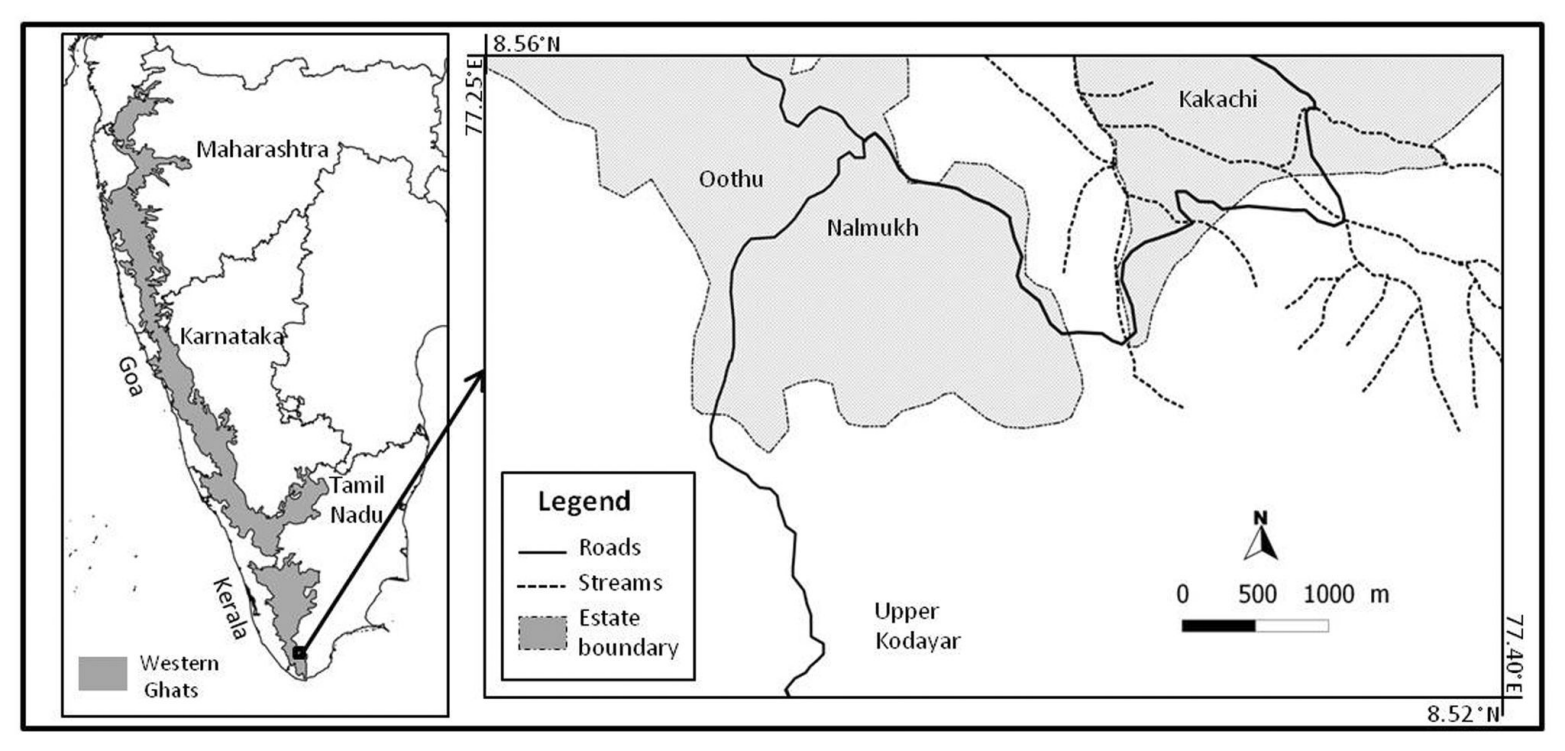
Map of the study site in the Western Ghats, and the Kakachi and Nalmukh tea estates of the Kalakad Mundanthurai Tiger Reserve. In the insert map, white area indicates forests and grey area indicates tea plantations with windbreaks.

Our study was carried out within the natural windbreaks in tea-plantations of Nalmukh and in the surrounding primary forests at Kakachi (8^o^32’ N and 77^o^21’ E; 1300 m above sea level), Kalakad Mundanthurai Tiger Reserve (hereafter ‘KMTR’), Southern Western Ghats, India (Figure 1), during the dry season from 18–20 August 2010 when migrants were absent. Mean annual rainfall is approximately 3000 mm and temperatures range between 19^o^C and 24^o^C in the mid-elevation wet-evergreen rainforests at the study site [21,22]. The rainforests at the study site are dominated by *Cullenia* *exarillata* , *Aglaia* *eleaegnoidea* and *Palaquium* *ellipticum* [16]. The tea plantations of the Bombay Burmah Trading Corporation (BBTC) cover a total area of 34km^2^ at the study site and dominate the landscape in Nalmukh [23]. This study was conducted with due permits from Forest Department and Electricity Board of Tamil Nadu, India.

The two habitat types compared were primary forests and natural windbreaks. The primary forests are continuous undisturbed old-growth interior rainforest sites in Kakachi (Figure 1). The windbreaks are 50-125 m wide linear fragments of natural vegetation that have been left between tea plantations in Nalmukh. These windbreaks were spread across the tea growing landscape of KMTR and connect the forest around the periphery of the plantations. Three different windbreaks were sampled during the study, which were at distances of between 100 and 500 m from the surrounding rainforest and were pooled for analysis as they did not show differences in bird species composition (R^2^ = 0.27, d.f. = 43, *P* = 0.88; see text in the analysis of habitat types for statistical details).

Both habitat types (windbreaks and primary forests) had similar tree density, diameter at breast height (DBH; 1.3m) of living trees ≥10 cm and tree height (online supplementary material, Supplementary Table S1). However, the DBH of the widest tree and height of the tallest tree in primary forest vegetation plots was significantly higher than that in windbreaks, confirming their lack of mature primary forest trees (see Supplementary Table S1 for details).

### Bird Sampling

The bird assemblages in primary forests and windbreaks were sampled using a fixed-width point count [24] between 6:30 and 10: 30hrs in the morning and 15:30 and 17: 30hrs in the afternoon for three days (18-20 August 2010). Each count was taken for ten minutes, followed by an interval of ten minutes during which the observer walked on a relatively straight line to the next point ~300m away. In each of the windbreaks at transect comprised 17 point counts and thus covered a distance of >5 km and was counted on one day. The transect in the primary forest comprised 51 points and thus extended for >15 km. Sampled points were surveyed only once, so that we did not inadvertently count the same individual more than once. This approach maximised the spatial independence of successive sample points. All bird detections (sighting and aural) within a 30m distance from a sampling station were recorded during each point count. The species and numbers of detections of each species were noted during each count. All over-flying birds and uncertain identifications were removed from the analysis. The study was conducted in August, the breeding season of most birds in the region, over three days with 51-point counts in each habitat type, amounting to a total of 1020 minutes of observation. All the 102 points through out the study site were broadly comparable in terms of geology, rainfall, climate and topography and forest structure (Supplementary Table S1). All bird sampling was carried out by A. Mohan and S. Das, who have over two years experience of identifying birds in Western Ghats by sight and calls. Observer A. Mohan surveyed the windbreaks for two days and primary forests for one day, and observer S. Das surveyed primary forests for two days and windbreaks for one day. This method helped us survey both the habitat types at the same time and sample greater number of points (51) in each habitat in a short period of time.

Data analyses

### Assessment of sampling effort

The species richness in each assemblage was calculated by computing species accumulation curves. We did not compute rarefaction curves because the number of points sampled in each habitat was equal (51), and the number of individuals detected at each habitat was nearly equal, 444 individuals in primary forest and 441 individuals in windbreaks. Therefore, the rarefaction curves are equal to the randomised original curves. To evaluate the effectiveness of sampling effort, the original bird species richness was transformed to an estimated richness by randomly adding 50 sampling sessions to the original data by using the bootstrap estimator, a measure that is considered more robust than other analytical estimators [25]. We used a regression model to estimate the correlation between the randomised original and bootstrap estimator data [26,27].

### Analysis of habitat types

Differences in abundance and richness between primary forests and windbreaks were estimated using a two-sample t-test after log_10_ transforming the data, to account for non-normality and heterogeneity of variances. To model the differences in community composition between habitat types, we used a multivariate generalised linear model on species count data [28]. We included observer (categorical: A. Mohan or S. Das) and habitat type (categorical: primary forest or windbreak) as predictor variables. Observer was incorporated to investigate for the possibility of observer bias. As we found no support for observer effects (*P* = 0.157), it was removed from the model during model simplification [29].

Multivariate generalised linear models provide a powerful framework for analysing species abundance data and have been shown to be more robust than distance based methods, such as multidimensional scaling and redundancy analysis [30,31]. Negative binomial regression was specified in our model, as our count data was over-dispersed [28,32]. Significance was assessed using 999 permutations of a Monte Carlo test. Species with less than four detections were removed from the community composition analysis to reduce the influence of accidental occurrences [33]. Analyses were carried out in R 2.14.2 [34]. We used package *Vegan* for calculating species richness and species accumulation curves [35], and package *mvabund* for testing the hypothesis about the community composition [28]. We used non-metric multidimensional scaling (NMDS) to visualise composition data.

### Analysis of Western Ghats endemics and functional groups

For each bird species recorded during the study period, we collated information on distribution (Western Ghats endemics or widely distributed) and dietary traits (vertebrates, invertebrates, fruits, grains and nectar) using Ali and Ripley [36] and Rasmussen and Anderton [37], supplemented by field observations. We then classified birds into three feeding guilds: frugivorous, insectivorous and nectarivorous. Carnivorous and granivorous birds were not included in the analysis as these feeding guild classes had only three and one species respectively (Supplementary Table S2). Again, birds with less than four detections were removed from the dataset prior analysis.

To model the differences in abundances between habitat types for each Western Ghats endemic bird species, we used generalised linear models with quasi-poisson errors and log links. To test the resilience of important functional groups to differences between habitat types, we used a non-parametric Wilcoxon rank sum test that is more suitable for small sample sizes and hierarchical data.

## Results

We recorded 885 individuals from 462 detections at 102 points across habitat types (primary forests and windbreaks) in the uplands of KMTR (Supplementary Table S2 Supplementary Table S3). Consequently, 39 (raw species richness; 66%) of the 59 resident bird species (excluding swifts and swallows as the method we used was not appropriate to sample these groups) known from the area [38] were used in the analysis. Although the study period is relatively brief, sampling across points seemed to be sufficient, at least on a relative basis, as estimated raw species richness was only slightly higher than observed richness (mean percentage increase in site richness with bootstrap estimator = 8.5±3.4%). The difference between estimated and observed raw species richness varied little between primary forests and windbreaks (average difference between habitat types = 4.1%). Moreover, the randomised original and the bootstrap estimator data were highly correlated for both primary forests (R^2^ = 0.995) and windbreaks (R^2^ = 0999). Hence, we made further direct comparisons with original species richness data rather than the estimated values.

**Figure 2:**
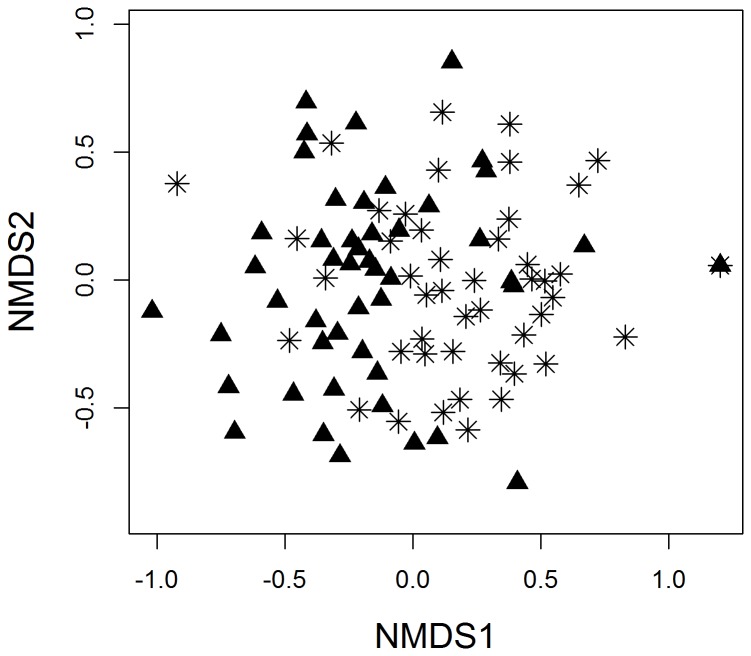
Non-metric multidimensional scaling (NMDS) ordination of the bird assemblages in primary forests (solid triangles) and windbreaks (asterisk). Points are census sites. The ordination diagram is for visualisation only; all tests of treatment effects are conducted using *mvabund* (see text for statistical details).

**Figure 3:**
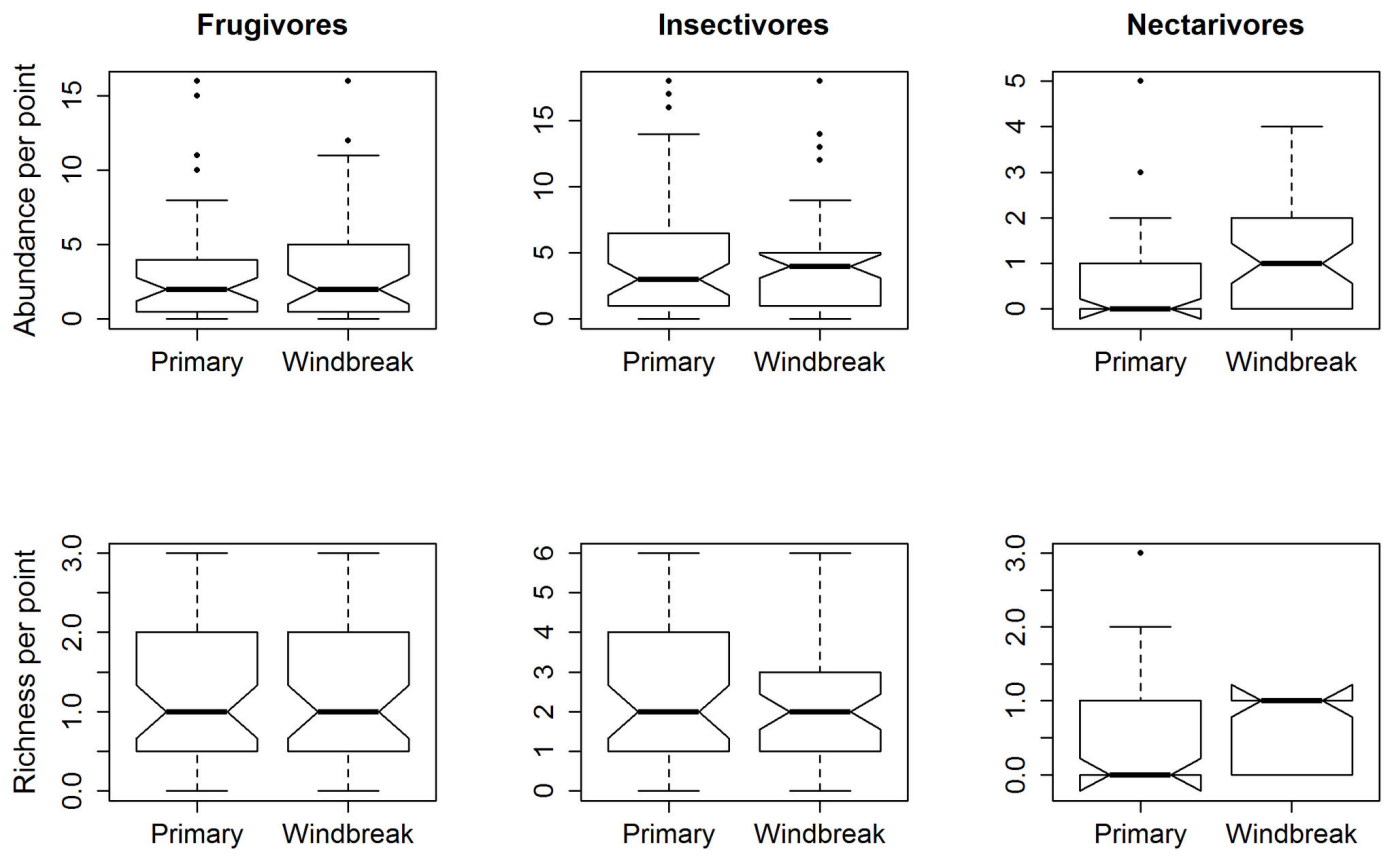
Notched box-plots showing the resilience of guild structure (abundance and richness) between primary forests and windbreaks. If the notches in the box plots do not overlap, you can conclude with 95% confidence that the true medians do differ. Plots show that nectarivorous birds were more resilient in windbreaks (Supplementary Table S4).

The observed raw species richness and total abundance of species per point were similar between primary forests and windbreaks (Table 1). However, the habitat types differed in their community composition (R^2^ = 0.22, d.f. = 93, *P* = 0.001; Figure 2). Nevertheless, majority of species used in the analysis (75%) occurred in both the habitat types causing some degree of overlap between habitat types (Figure 2). We found ten bird species that are endemic to the Western Ghats (26% of the raw species richness). Of these eight occurred in primary forests and seven in windbreaks (Supplementary Table S2). Only five endemic species were included in the analyses because of low abundances of the other five species (Supplementary Table S2). Among the five endemic species used in the analysis, the abundances of Kerala Laughingthrush ( *Trochalopteronfairbanki* ) and Small Sunbird ( *Nectarinia* *minima* ) increased in windbreaks (Table 2), while there was no change in the abundances of the other three species between habitat types (Table 2). Nectarivorous birds increased in species richness and total abundance per point in windbreaks as compared to primary forest (Figure 3). There was no difference in total abundance and species richness per point of frugivorous and insectivorous birds between habitat types (Figure 3 Supplementary Table S4).

**Table 1:** Mean ± standard deviation of bird species richness, abundance in primary forests and windbreaks.

	**Primary**	**Windbreak**	**Total**
**Total species observed**	30	31	39
**Raw species richness per point**	4.14 ± 2.20^a^	3.92 ± 2.05^a^	4.03 ± 2.12
**Total abundance per point**	8.71 ± 7.26^a^	8.65 ± 6.09^a^	8.68 ± 6.67

Variables were log_10_ transformed. Letters denote significance under two-sample t-test (*P* ≤ 0.05), same letter indicates no significant differences among means.

**Table 2:** Responses of Western Ghats endemic and threatened species to natural windbreaks.

**Species**	**F**	***P***	**Response**
**Square-tailed Black Bulbul ( *Hypsipetes* *ganeesa* )**	0.64	0.43	NS
**Kerala Laughingthrush ( *Trochalopteronfairbanki* )**	17.85	<0.0001	positive
**Black-and-Orange Flycatcher ( *Ficedula* *nigrorufa* )**	0.06	0.81	NS
**Nilgiri Flycatcher ( *Eumyias* *albicaudatus* )**	0.21	0.65	NS
**Small Sunbird ( *Nectarinia* *minima* )**	6.66	0.01	positive

F and *P* values are derived from the generalised linear model with quasi-poisson errors and log link. Positive response indicates increase in abundance in natural windbreaks and NS indicates insignificant effect.

## Discussion

The high conservation value of natural windbreaks that contain remnant vegetation and facilitate the use and movement of birds between fragments of forest has not yet been sufficiently highlighted [8]. Our study shows that a rich pool of birds makes use of natural windbreaks. We detected 80% of the overall bird species pool in windbreaks including seven of the ten-recorded Western Ghats endemics. In particular, the Kerala Laughingthrush ( *Trochalopteronfairbanki* ) and Small Sunbird ( *Nectarinia* *minima* ) appear to use natural windbreaks frequently.

It has been suggested that windbreaks with native vegetation like those in our study site serve as habitats and nesting sites for birds, whereas planted windbreaks (e.g., Eucalyptus) may only be transient foraging sites and travel paths [8]. A three-year study of corridor use by birds in Costa Rica found that the bird species richness was higher in natural windbreaks than in planted windbreaks [7,8]. Our study extends the earlier investigations on the use of natural windbreaks by birds in the tropics by comparing the species richness and abundances of assemblages in windbreaks with that found in the primary forests of Western Ghats.

We found that bird species richness was similar in primary forests and windbreaks. One of the reasons for such similarity might be the rainforest dominated landscape of the study area [21]. Spill-over effects may exaggerate the species richness of windbreaks, since windbreaks in Nalmukh are contiguous with rainforest and surrounded by it [39,40]. Though habitat types showed similarities in bird species richness and abundance, the community composition was different, perhaps because most forest edge species such as the Red-whiskered Bulbul ( *Pycnonotus* *jocosus* ), Common Iora ( *Aegithina* *tiphia* ), Black-throated Munia ( *Lonchura* *kelaarti* ) and Indian Rufous Babbler ( *Turdoides* *subrufa* ) benefited from the presence of windbreaks. When windbreaks connect forests, they form complex, heterogeneous habitats that may increase bird species richness and abundance [8].

Natural windbreaks also enhance the richness and abundance of ecological service providers such as frugivores, insectivores and nectarivores against extensive species loss in tea plantations [13]. Windbreaks benefit from higher amounts of light-demanding tree species and increased flower and fruit production in gaps and edges. Windbreaks in our study site had native secondary forest fruiting and flowering trees like *Elaeocarpus* *serratus* *,* *Elaeocarpus* *munronii* and *Persea* *macrantha* that have drupaceous bird-dispersed fruit. This enables frugivorous and nectarivorous birds to use them as regular foraging sites as such species typically travel long distances between food resources and have lower fragmentation sensitivity than insectivores [41,42].

There was no difference in species richness or abundances of insectivores between primary forests and windbreaks at our study site (Figure 2). Therefore, they do not appear to be especially sensitive to natural windbreaks, although insectivores have often been reported as being sensitive to disturbance or edge habitats [4,41]. Moreover, one of us (R. Vivek) has been studying bird communities in this area and has observed many forest birds nesting in windbreaks, including the IUCN near-threatened Western Ghats endemic insectivores such as Black and Orange Flycatcher ( *Ficedula* *nigrorufa* ) and the Nilgiri Flycatcher ( *Eumyias* *albicaudatus* ) [43]. Hence, natural windbreaks may not just act as travel routes, but also as foraging and nesting sites for forest birds. Future studies that focus on the habitat use, movement, foraging and nesting patterns of forest birds in natural windbreaks over a long time period will generate critical insights into the importance of natural windbreaks in production landscapes.

### Implications for conservation

According to our results, natural windbreaks appear to be important habitats that may sustain bird species richness and abundance in production landscapes. They also have similar frugivore species richness and abundance as primary forests, which can help disperse seeds into adjoining tea plantations and therefore aid regeneration or possibly between forest patches [44]. A recent study in our study area examined the potential value of shade trees in tea plantations for seed dispersal and found that the density of shade trees has a strong influence on seed arrival [16]. Thus, tea plantations with natural windbreaks that connect fragments and maintain greater density of native shade trees should be supported by, for example, setting premium prices for biodiversity-friendly tea production.

### Limitations and directions to future research

Some caution is required while interpreting our results as the study was conducted during a brief period using multiple observers. The short duration of the study means we fail to capture any seasonal variation in the importance of windbreaks. An increased duration of sampling might also reveal that some species, which we found to be restricted to one habitat type, may actually be present in both the habitat types. For example, Oriental White-eye ( *Zosterops* *palpebrosus* ) was only recorded in the windbreaks, but they were regularly sighted in primary forests before and after the study period (R. Vivek, pers. obs*.*). Although our study provides a valuable preliminary insight into the use of windbreaks by birds, we highlight the need for additional studies. We recommend investigating a wider range of organisms and different windbreak types (shape, vegetation and distance from forest) to understand how to effectively manage and conserve biodiversity in this landscape.

## Supporting Information

Table S1 Mean ± standard deviation for vegetative characteristics of primary forests and natural windbreaks in the uplands of KMTR. Differences among habitat types were tested with two-sample t-tests after 1og_**10**_ transforming the variables. Data was obtained from 20 randomly placed 10 x 5m plots in each habitat type. To avoid disturbances, the vegetation was sampled after all bird sampling was completed.
(DOC)

Table S2 Total species abundance in primary forests and windbreaks. Feeding guild codes are: C – carnivore, F – frugivore, G – granivore, I – insectivore and N – nectarivore. Status codes are: * – IUCN Red Data book status (threatened or above), + – endemic to Western Ghats, India.
(DOC)

Table S3 Species detected at each point. Points starting with 'P' are primary forest and with 'D' are natural windbreaks. UTM data available on request from the corresponding author.
(XLS)

Table S4 Effects of natural windbreaks on bird guild resilience. W and *P* values are derived from Wilcoxon rank sum test. Positive response indicates increase in richness or abundance in natural windbreaks and NS indicates insignificant effect.
(DOC)
