# Supplementary material for: Natural Windbreaks Sustain Bird Diversity in a Tea-Dominated Landscape
Source: PLoS One. 2013 Jul 29;8(7):e70379. doi: 10.1371/journal.pone.0070379 (PMC3726631; doi:10.1371/journal.pone.0070379)
Supplement: Table S1 — Differences among habitat types were tested with two-sample t-tests after 1og10 transforming the variables. Data was obtained from 20 randomly placed 10 x 5m plots in each habitat type. To avoid disturbances, the vegetation was sampled after all bird sampling was completed. (DOC) [file pone.0070379.s001.doc]

**Supplementary Table S1. Mean ± standard deviation for vegetative characteristics of primary forests and natural windbreaks in the uplands of KMTR.** Differences among habitat types were tested with two-sample t-tests after 1og_10_ transforming the variables. Data was obtained from 20 randomly placed 10 x 5m plots in each habitat type. To avoid disturbances, the vegetation was sampled after all bird sampling was completed.

|  | **Primary forest** | **Windbreak** | **t** | ***P*** |
| --- | --- | --- | --- | --- |
| **Number of trees per plot** | 6.5 ± 2.52 | 5.6 ± 2.35 | 1.205 | 0.235 |
| **Tree height (m)** | 16.51 ± 10.28 | 13.62 ± 5.33 | 1.352 | 0.178 |
| **DBH (m)** | 0.26 ± 0.21 | 0.23 ± 0.16 | 0.724 | 0.470 |
| **Height of the tallest tree in a plot(m)** | 31.29 ± 8.26 | 19.92 ± 5.28 | 4.824 | <0.001 |
| **DBH of the widest tree in a plot (m)** | 0.64 ± 0.21 | 0.42 ± 0.27 | 3.764 | <0.001 |
