# Supplementary material for: Natural Windbreaks Sustain Bird Diversity in a Tea-Dominated Landscape
Source: PLoS One. 2013 Jul 29;8(7):e70379. doi: 10.1371/journal.pone.0070379 (PMC3726631; doi:10.1371/journal.pone.0070379)
Supplement: Table S2 — Feeding guild codes are: C – carnivore, F – frugivore, G – granivore, I – insectivore and N – nectarivore. Status codes are: * – IUCN Red Data book status (threatened or above), + – endemic to Western Ghats, India. (DOC) [file pone.0070379.s002.doc]

**Supplementary Table S2. Total species abundance in primary forests and windbreaks.** Feeding guild codes are: C – carnivore, F – frugivore, G – granivore, I – insectivore and N – nectarivore. Status codes are: * – IUCN Red Data book status (threatened or above), + – endemic to Western Ghats, India.

| **Species** | **Feeding Guild** | **Primary**  **forests** | **Windbreaks** | | **Status** |
| --- | --- | --- | --- | --- | --- |
| **Crested Serpent-Eagle (*Spilornis cheela*)** | C | 2 | 0 | - | |
| **Red Spurfowl (*Galloperdix spadicea*)** | I | 0 | 1 | - | |
| **Grey Junglefowl (*Gallus sonneratii*)** | I | 8 | 4 | * | |
| **Mountain Imperial-Pigeon (*Ducula badia*)** | F | 8 | 21 | - | |
| **Vernal Hanging Parrot (*Loriculus vernalis*)** | F | 1 | 3 | - | |
| **Greater Coucal (*Centropus sinensis*)** | C | 0 | 1 | - | |
| **Malabar Trogon (*Harpactes fasciatus*)** | I | 6 | 0 | - | |
| **White-cheeked Barbet (*Megalaima viridis*)** | F | 6 | 10 | - | |
| **Greater Flameback (*Chrysocolaptes lucidus*)** | I | 4 | 5 | - | |
| **Orange Minivet (*Pericrocotus flammeus*)** | I | 2 | 1 | + | |
| **Pied Flycatcher-shrike (*Hemipus picatus*)** | I | 18 | 0 | - | |
| **Red-whiskered Bulbul (*Pycnonotus jocosus*)** | F | 0 | 20 | - | |
| **Yellow-browed Bulbul (*Iole indica*)** | F | 74 | 25 | - | |
| **Square-tailed Black Bulbul (*Hypsipetes ganeesa*)** | F | 76 | 96 | + | |
| **Common Iora (*Aegithina tiphia*)** | I | 0 | 2 | - | |
| **Malabar Whistling-thrush (*Myiophonus horsfieldii*)** | I | 25 | 13 | - | |
| **Orange-headed Thrush (*Zoothera citrina*)** | I | 2 | 1 | - | |
| **Indian Blackbird (*Turdus simillimus*)** | I | 7 | 28 | - | |
| **White-bellied Shortwing (*Brachypteryx albiventris*)** | I | 2 | 0 | *, + | |
| **Kerala Laughingthrush (*Trochalopteron fairbanki*)** | I | 0 | 16 | *, + | |
| **Indian Scimitar-babbler (*Pomatorhinus horsfieldii*)** | I | 31 | 18 | - | |
| **Dark-fronted Babbler (*Rhopocichla atriceps*)** | I | 41 | 3 | - | |
| **Indian Rufous Babbler (*Turdoides subrufa*)** | I | 0 | 6 | + | |
| **Brown-cheeked Fulvetta (*Alcippe poioicephala*)** | I | 24 | 16 | - | |
| **Large-billed Leaf-warbler (*Phylloscopus magnirostris*)** | I | 1 | 1 | - | |
| **Black-and-Orange Flycatcher (*Ficedula nigrorufa*)** | I | 7 | 8 | *, + | |
| **Nilgiri Flycatcher (*Eumyias albicaudatus*)** | I | 3 | 2 | *, + | |
| **Grey-headed Canary-flycatcher (*Culicicapa ceylonensis*)** | I | 33 | 15 | - | |
| **Indian Yellow Tit (*Parus aplonotus*)** | I | 16 | 6 | - | |
| **Velvet-fronted Nuthatch (*Sitta frontalis*)** | I | 9 | 0 | - | |
| **Thick-billed Flowerpecker (*Dicaeum agile*)** | N | 2 | 0 | - | |
| **Nilgiri Flowerpecker (*Dicaeum concolor*)** | N | 1 | 0 | + | |
| **Purple-rumped Sunbird (*Leptocoma zeylonica*)** | N | 0 | 2 | - | |
| **Small Sunbird (*Nectarinia minima*)** | N | 18 | 45 | + | |
| **Little Spiderhunter (*Arachnothera longirostra*)** | N | 6 | 1 | - | |
| **Oriental White-eye (*Zosterops palpebrosus*)** | I | 0 | 66 | - | |
| **Black-throated Munia (*Lonchura kelaarti*)** | G | 0 | 3 | - | |
| **Greater Racket-tailed Drongo (*Dicrurus paradiseus*)** | I | 8 | 2 | - | |
| **White-bellied Treepie (*Dendrocitta leucogastra*)** | C | 3 | 0 | + | |
