# Supplementary material for: Natural Windbreaks Sustain Bird Diversity in a Tea-Dominated Landscape
Source: PLoS One. 2013 Jul 29;8(7):e70379. doi: 10.1371/journal.pone.0070379 (PMC3726631; doi:10.1371/journal.pone.0070379)
Supplement: Table S4 — W and P values are derived from Wilcoxon rank sum test. Positive response indicates increase in richness or abundance in natural windbreaks and NS indicates insignificant effect. (DOC) [file pone.0070379.s004.doc]

**Supplementary Table S4. Effects of natural windbreaks on bird guild resilience.** W and *P* values are derived from Wilcoxon rank sum test. Positive response indicates increase in richness or abundance in natural windbreaks and NS indicates insignificant effect.

|  | **Abundance** | | | **Species Richness** | | |
| --- | --- | --- | --- | --- | --- | --- |
|  | W | *P* | Response | W | *P* | Response |
| **Frugivores** | 1282.5 | 0.905 | NS | 1268.5 | 0.823 | NS |
| **Insectivores** | 1328.5 | 0.852 | NS | 1483.5 | 0.211 | NS |
| **Nectarivores** | 1046 | 0.047 | positive | 1007 | 0.026 | positive |
